# Mathematical Model for Estimating Parameters of Swelling Drug Delivery Devices in a Two-Phase Release

**DOI:** 10.3390/polym12122921

**Published:** 2020-12-05

**Authors:** Amanina Setapa, Naveed Ahmad, Shalela Mohd Mahali, Mohd Cairul Iqbal Mohd Amin

**Affiliations:** 1 Faculty of Ocean Engineering Technology & Informatics, Universiti Malaysia Terengganu, Kuala Nerus 21030, Malaysia; p3189@pps.umt.edu.my; 2 Department of Pharmaceutics, College of Pharmacy, Jouf University, Sakaka 72388, Saudi Arabia; naveedpharmacist@yahoo.com; 3 Management Sciences Research Group, Universiti Malaysia Terengganu, Kuala Nerus 21030, Malaysia; 4 Centre of Drug Delivery Research, Faculty of Pharmacy, University Kebangsaan Malaysia, Jalan Raja Muda Abdul Aziz, Kuala Lumpur 50300, Malaysia; mciamin@ukm.edu.my

**Keywords:** swelling hydrogels, two-phase drug release, constant diffusion coefficient

## Abstract

Various swelling drug delivery devices are promising materials for control drug delivery because of their ability to swell and release entrapped therapeutics, in response to physiological stimuli. Previously, many mathematical models have been developed to predict the mechanism of drug release from a swelling device. However, some of these models do not consider the changes in diffusion behaviour as the device swells. Therefore, we used a two-phase approach to simplify the mathematical model considering the effect of swelling on the diffusion coefficient. We began by defining a moving boundary problem to consider the swelling process. Landau transformation was used for mitigating the moving boundary problem. The transformed problem was analytically solved using the separation of variables method. Further, the analytical solution was extended to include the drug release in two phases where each phase has distinct diffusion coefficient and continuity condition was applied. The newly developed model was validated by the experimental data of bacterial cellulose hydrogels using the LSQCURVEFIT function in MATLAB. The numerical test showed that the new model exhibited notable improvement in curve fitting, and it was observed that the initial effective diffusion coefficient of the swelling device was lower than the later effective diffusion coefficient.

## 1. Introduction

The safe and effective delivery of therapeutics at the target site of action is a challenging task for drug delivery scientists. High dose or dosage frequency, undesirable variations in drug concentration, nonspecific distribution of drugs, and severe side effects are the major limitations associated with conventional drug administration. These limitations lead to poor patient compliance and therapeutic response [1,2,3]. To overcome these limitations, controlled release drug delivery devices, such as those using hydrogels, nanoparticles, micelles, liposomes, and membranes, have gained considerable attention in the last four decades [4]. These controlled release systems are designed to deliver drugs at the desired rate to the target site; thus, they can reduce the dosage and toxicity of a drug and enhance drug efficacy [2]. Stimuli-responsive materials that exhibit conformational changes in their structure in response to physical or physiological stimuli, such as pH, temperature, ionic strength, biomolecules (enzymes), light, redox potential, and electric/magnetic field, are widely explored to trigger the release of drugs at the target site [5,6]. Drug delivery devices that exhibit changes in their swelling behaviour in response to various stimuli, such as hydrogels, are promising as drug delivery applications.

Among the various drug delivery devices used for controlled release, hydrogels represent one of the most dynamic classes of drug delivery systems. Hydrogels are broadly defined as ‘three-dimensional (3D) polymer networks that can retain a large amount of water in their swollen state’ [7,8,9]. Over the last fifty years, hydrogels have become one of the most popular materials for various applications [7]. They have been employed for controlled delivery of drugs, therapeutic proteins, genes, stem cells, immunoglobulins, and vaccines via different routes of administration to various tissue, because of their swelling behaviour in an aqueous environment, biocompatibility, biodegradability, and non-toxicity [10,11,12]. Stimuli-responsive swelling is the most important attribute of the hydrogels that are exploited for controlled release. For instance, hydrogels have been investigated for the oral delivery of therapeutic proteins because of their pH-responsive swelling behaviour that allows the hydrogel to hold the release in the stomach (lower swelling at acidic pH) and release the protein in the small intestine (higher swelling at neutral pH) [3,7,9,11,12].

Mathematical models assist in predicting the rate, extent, and mechanism of drug release from a delivery device, thereby accelerating the process of designing optimal pharmaceutical formulation by reducing the number of required experiments [13]. The release mechanisms of a delivery device are mainly categorised into diffusion, swelling/dissolution, and chemically and osmotically controlled. However, in hydrogel-based drug delivery devices, the diffusion and swelling phenomena occur concurrently and may be followed or accompanied by polymer degradation and/or dissolution under suitable conditions [13,14,15]. Therefore, modelling of drug release from hydrogel-based delivery devices becomes more challenging when considering these phenomena simultaneously. Numerous mathematical models have been presented for hydrogel-based drug delivery devices, which have been reviewed comprehensively in other studies [13,14,16].

The development of these drug release models began with Fick’s law of diffusion, and then other phenomena, such as swelling, polymer degradation, the microstructure of hydrogel, glassy/rubbery shifts, the geometry of the drug delivery device, and stimuli response, were considered in these mathematical models [13]. Fick’s law of diffusion is a model that describes the dependence of drug diffusion rate on drug concentration function and can be used to quantify the drug transport in a device [17]. Brazel and Peppas extended Fick’s law to include the polymer relaxation rate while estimating swelling-controlled release [14]. Furthermore, Peppas et al. introduced an empirical equation by assuming a time-dependent power law function, which is widely used in the study of drug release owing to its simplicity [13]. This model helps in distinguishing the different release mechanisms of a device that can be swelling-controlled, diffusion-controlled, or anomalous transport. However, the power law model is only suitable for estimating drug release under 60% of the whole release process [13,18]. Besides, both Fick’s law and power law model cannot determine the swelling behaviour of a device.

The advection–diffusion equation is another model that can represent drug diffusion and hydrogel swelling simultaneously [18,19]. Unlike Fick’s law and power law model, this equation can determine the swelling behaviour of a device since this equation includes a growth velocity function. Bierbrauer used the advection–diffusion equation to explain drug diffusion in swelling devices and Landau transformation to overcome the complexity of the model caused by the swelling behaviour [20]. In this study, we applied a similar approach to solve the drug delivery problem of swelling devices that involves non-homogeneous boundary conditions for a non-sink condition [21]. Recently, Kalkhoran et al. [22] introduced a new mathematical approach and developed a drug release model from a highly swellable device by introducing a new parameter into a nonlinear equation of diffusion coefficient. This model efficiently predicted the maximum release of drugs from the swelling device but no analytical solution was given in this article.

The research work on mathematical modelling of controlled drug delivery has been significantly advanced; however, the effect of swelling on the diffusion coefficient is usually neglected in the mathematical model. The diffusion coefficient is a significant parameter in estimating the drug release, especially in a diffusion-controlled release device as the diffusion rate depends on this parameter [23]. The diffusion coefficient is also known as the diffusivity or the ability of a substance to diffuse. The diffusion coefficient can be in a form of a constant or a function of time. Most researchers tend to consider the diffusion coefficient as a constant parameter [17,18,19,20,21]. However, for a device with a significantly high swelling rate, a constant diffusion coefficient might not relevant. A study by Fu et al. [24] showed that, as the swelling ratio of a hydrogel increases, the diffusion ratio of the sample also increases. Theoretically, as the device swells, the pore size gets larger, facilitating the drug diffusion and, eventually, causing an increment in the diffusion coefficient. The study by Ahmad et al. [9] showed that the pore size of a higher swelling device is bigger than devices with lower swelling. The diffusion–porosity relation computed by Ray et al. [25] shows that the diffusion differs with different porosity values and geometries.

Therefore, it is worth developing a mathematical model that acknowledges the dynamic behaviour of drug diffusion as the drug device swells. However, in some conditions, considering the diffusion coefficient as a function of time will not give an analytical solution [26]. Previously, to study the dynamic behaviour of drug diffusion during the initial burst release, Wang et al. [27] divided the whole drug release process into two phases with distinct constant diffusion coefficient in each phase. The first phase is the initial burst release phase and the second phase is the normal release phase. Therefore, in this study, we developed a mathematical model for estimating drug release from a swelling hydrogel-based drug delivery device considering the dynamical constant diffusion coefficient by adopting the idea of two-phase drug release. We solved the fractional drug release model using the advection–diffusion equation, an approach similar to that used by Bierbrauer [20]. The solved fractional drug release model by Bierbrauer [20] was introduced as a one-phase model in this study. Further, the model was extended to include the two-phase release by splitting the drug release process into two phases, as suggested by Wang et al. [27]. Both phases have constant diffusion coefficients with different values.

## 2. Mathematical Solution for Drug Release from a Swelling Device

**Figure 1:**
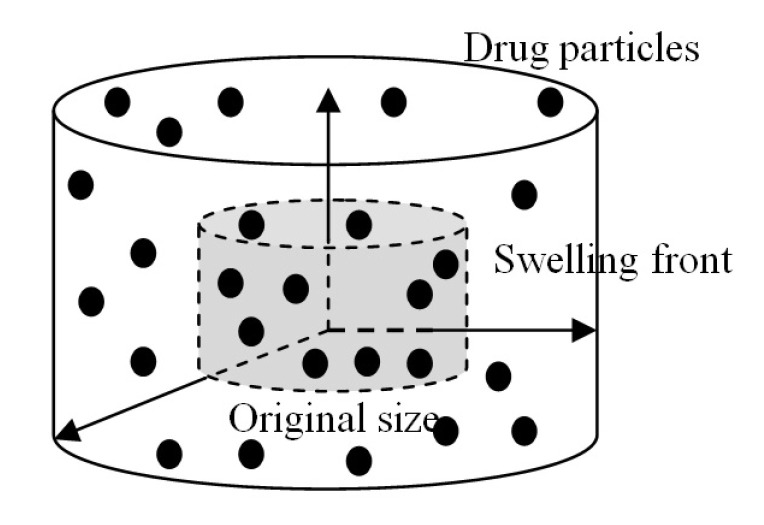
Illustration of drug diffusion and device swelling in a 3D space.

The drug release from a swelling device to an external fluid is influenced by many factors, such as drug diffusion and device swelling processes. The diffusion and swelling of a cylindrical drug delivery device (hydrogel disc) in a 3D space are illustrated in Figure 1.

In this section, two fractional drug release models for swelling devices are developed: one-phase and two-phase models. The one-phase model represents a drug release with a single constant diffusion coefficient throughout the entire release process while the two-phase model represents a drug release with a piecewise diffusion coefficient.

### 2.1. One-Phase Model

We start from a basic model considering a single constant diffusion coefficient. The advection–diffusion equation is a well-known model when considering the drug diffusion and device swelling problem simultaneously. The general advection–diffusion equation is expressed as

$$\frac{\partial c}{\partial t}=▽\cdot \left(D(t)▽c\right)-▽\cdot \left(cu\right),$$

where $\frac{\partial c}{\partial t}$ is the local rate change of concentration over time, c(x,y,z,t) is the concentration of drug in the device, D(t) is diffusion coefficient, ▽c is the concentration gradient, and u(x,y,z,t) is the device swelling velocity. To solve this equation analytically, the diffusion coefficient is assumed as a constant, where ▽D≡0. Thus,

(1) $$\frac{\partial c}{\partial t}=D{▽}^{2}c-▽\cdot \left(cu\right).$$

**Figure 2:**
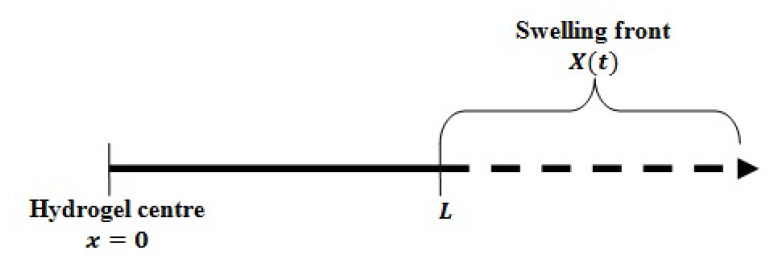
Illustration of the 1D region.

For simplicity, the one-dimensional (1D) case with a sink boundary condition is considered. The 1D region of device swelling and drug diffusion is shown in Figure 2.

The 1D case model of drug release from a swelling device is then expressed as

(2) $$\frac{\partial c}{\partial t}=D\frac{\partial^{2}c}{\partial x^{2}}-u\frac{\partial c}{\partial x}-c\frac{\partial u}{\partial x}\;\mathrm{in}\;0<x<X\left(t\right),t>0,$$

with a constant initial condition $c\left(x,0\right)=c_{0}$ in 0<x<X(t). No flux boundary conditions were assumed at the left edge (hydrogel centre), $\frac{\partial c}{\partial x}\left(0,t\right)=0$, and the sink boundary condition was assumed at the right edge (hydrogel surface/boundary), c(X(t),t)=0. The boundary X(t) is moving only in positive *x* direction starting from X(0)=L. The device swelling is assumed to be uniform.

As the device swells, the velocity gradient is located across the region of the device. The velocity gradient is the difference in the velocities of growth (growing) and initial boundaries. This relation can be expressed as

$$u\left(X(t),t\right)-u\left(0,t\right)=\int_{0}^{X(t)}\frac{\partial u}{\partial x}dx=\frac{dX(t)}{dt}.$$

Therefore, the velocity, *u* is expressed as

(3) $$u\left(x,t\right)=\frac{\dot{X}\left(t\right)}{X(t)}x.$$

Using Equation (3), Equation (2) can be rewritten as

(4) $$\frac{\partial c}{\partial t}=D\frac{\partial^{2}c}{\partial x^{2}}-\left(\frac{\dot{X}\left(t\right)}{X(t)}x\right)\frac{\partial c}{\partial x}-c\frac{\dot{X}\left(t\right)}{X(t)}\;\mathrm{in}\;0<x<X\left(t\right),t>0,$$

with the following initial and boundary conditions:

$$\begin{array}{c} c\left(x,0\right)=c_{0}\;\;\mathrm{in}\;\;0<x<X\left(t\right),\\ \frac{\partial c}{\partial x}\left(0,t\right)=0,t>0,\\ c(X(t),t)=0,t>0. \end{array}$$

As the moving boundary domain of the above model is difficult to solve, Landau transformation is applied in the model, wherein the new parameters are defined as

$$\zeta =\frac{x}{X(t)},\;\tau =t.$$

Introducing the parameter ζ not only helps in handling the original moving boundary problem but also eliminates the advection term, thereby providing a simpler model to represent the drug transport in a swelling device.

(5) $$\frac{\partial c}{\partial \tau }=\frac{D}{X{\left(t\right)}^{2}}\frac{\partial^{2}c}{\partial \zeta^{2}}-\frac{\dot{X}\left(t\right)}{X(t)}c\;\mathrm{in}\;0<\zeta <1,\tau >0,$$

with the following initial and boundary conditions:

$$\begin{array}{c} c\left(\zeta ,0\right)=c_{0}\;\;\mathrm{in}\;\;0<\zeta <1,\\ \frac{\partial c}{\partial \zeta }\left(0,\tau \right)=0,\tau >0,\\ c(1,\tau )=0,\tau >0. \end{array}$$

After the transformation, the spatial domain of this model is a constant between 0 and 1, instead of a moving domain that depends on time. Equation (5) can be solved using the separation of variables method. The solution is assumed to be a product of the functions of ζ and τ,

c(ζ,τ)=A(ζ)B(τ).

From the separation of variables method, two sets of ordinary differential equations (ODE) are obtained:

(6) $$A^{\prime \prime }+A\lambda^{2}=0$$

and

(7) $$\dot{B}+\left(\frac{\dot{X}\left(t\right)}{X(t)}+\frac{\lambda^{2}D}{X{\left(t\right)}^{2}}\right)B=0.$$

Equations (6) and (7) are solved subject to the boundary conditions, giving

$$A\left(\zeta \right)=a_{2}\cos\frac{\left(2n+1\right)}{2}\pi \zeta$$

and

$$B\left(\tau \right)=\frac{CL}{X(\tau )}e^{-D\lambda^{2}\int_{0}^{\tau }X{\left(t\right)}^{-2}dt}.$$

Further, the solutions of A(ζ) and B(τ) are rearranged using superposition to get an equation of c(ζ,τ).

(8) $$c\left(\zeta ,\tau \right)=\sum_{n=0}^{\infty }d_{n}\frac{L}{X(\tau )}\cos\left(\frac{\left(2n+1\right)}{2}\pi \zeta \right)e^{-D{\left(\frac{2n+1}{2}\pi \right)}^{2}\int_{0}^{\tau }X{\left(t\right)}^{-2}dt}$$

The initial condition, $c\left(\zeta ,0\right)=c_{0}$, is then employed in the above equation, thereby resulting in

$$d_{n}=c_{0}\frac{4{\left(-1\right)}^{n}}{(2n+1)\pi }.$$

Substituting the solution of $d_{n}$ into Equation (8), the following equation is obtained:

(9) $$c\left(\zeta ,\tau \right)=\frac{4}{\pi }c_{0}\sum_{n=0}^{\infty }\frac{{\left(-1\right)}^{n}}{\left(2n+1\right)}\frac{L}{X(\tau )}\cos\left(\frac{\left(2n+1\right)}{2}\pi \zeta \right)e^{-D{\left(\frac{2n+1}{2}\pi \right)}^{2}\int_{0}^{\tau }X{\left(t\right)}^{-2}dt}.$$

The derived solution, Equation (9), is the equation of the concentration of drugs in the hydrogel. Further, an equation for fractional drug release is derived to study the release rate in a swelling device. Fractional drug release can be defined by

(10) $$R\left(\tau \right)=\frac{M(\tau )}{M(0)}=1-\frac{X(\tau )}{Lc_{0}}\int_{0}^{1}c(\zeta ,\tau )d\zeta ,$$

where M(τ) is the total mass released and M(0) is the total initial drug loaded. Total mass released, M(τ), can be calculated by subtracting the mass of drug remained in the hydrogel (at any time τ) from the total initial drug loaded, $M\left(\tau \right)=M\left(0\right)-\int_{0}^{1}c(\zeta ,\tau )X(\tau )d\zeta$. Substituting Equation (9) into Equation (10), we have

$$R\left(\tau \right)=1-\sum_{n=0}^{\infty }\frac{8}{{\left(2n+1\right)}^{2}\pi^{2}}e^{-D{\left(\frac{\left(2n+1\right)}{2}\pi \right)}^{2}\int_{0}^{\tau }X{\left(t\right)}^{-2}dt}.$$

In original variables, this equation can be defined as

(11) $$R\left(t\right)=1-\sum_{n=0}^{\infty }\frac{8}{{\left(2n+1\right)}^{2}\pi^{2}}e^{-D{\left(\frac{\left(2n+1\right)}{2}\pi \right)}^{2}\int_{0}^{t}X{\left(t^{\prime }\right)}^{-2}dt^{\prime }}.$$

The integral term in Equation (11) includes the growth boundary function, X(t), which represents the swelling behaviour. In this study, we consider the logistic growth function

(12) $$X\left(t\right)=\frac{Le^{gt}}{1+\left(\frac{1}{m}\right)\left(e^{gt}-1\right)},$$

where $m=\lim_{t\to \infty }\frac{X(t)}{L},\;L$ is the initial length of the drug device, and *g* is the growth parameter. This equation is then integrated and substituted into the fractional drug release equation, expressed in Equation (11). By solving the integration of the logistic function, the fractional drug release, Equation (11), can be expressed as

(13) $$R\left(t\right)=1-\sum_{n=0}^{\infty }\frac{8}{{\left(2n+1\right)}^{2}\pi^{2}}e^{-\frac{D}{2g}{\left(\frac{2n+1}{2Lm}\pi \right)}^{2}P\left(t\right)},$$

where

$$P\left(t\right)={\left(m-1\right)}^{2}\left(1-e^{-2gt}\right)+4\left(m-1\right)\left(1-e^{-gt}\right)+2gt.$$

The solution for the fractional drug release equation, Equation (13), represents the one-phase model.

### 2.2. Two-Phase Model

Now, we extend the one-phase model to the two-phase model. The two-phase model is important to acknowledge the fact that the diffusion coefficient for a swelling drug device cannot remain constant throughout the process. The changes in the device structure due to swelling will affect the drug diffusivity through the device. Theoretically, as the device swells, the device porosity increases, and the diffusion coefficient is also increased. Instead of considering the general D=D(t), our approach is to divide the whole release process into two phases. The constant *D* differs in each phase

$$D=\left\{\begin{array}{c} D_{0},\;0<t<t_{c},\\ D_{1},\;t\geq t_{c}, \end{array}\right.$$

where $D_{0}$ and $D_{1}$ are the diffusion coefficients in the first and second phases. The critical time, $t_{c}$, is the borderline between the first and second phases of the entire release profiles. In developing this two-phase drug release model, the growth parameter, *g*, is assumed to be constant throughout the entire release process.

As similar methods from solving the one-phase model are to be adapted in this two-phase model, we begin by applying the transformed advection–diffusion equation, Equation (5), in two different phases with two different parameters of diffusion coefficients.

$$\frac{\partial c}{\partial \tau }=\{\begin{array}{ccc} \frac{D_{0}}{X{\left(t\right)}^{2}}\frac{\partial^{2}c}{\partial \zeta^{2}}-\frac{\dot{X}\left(t\right)}{X(t)}c, & 0<\tau <\tau_{c},\;\mathrm{in}\;0<\zeta <1, & (14a)\\ \frac{D_{1}}{X{\left(t\right)}^{2}}\frac{\partial^{2}c}{\partial \zeta^{2}}-\frac{\dot{X}\left(t\right)}{X(t)}c, & \tau \geq \tau_{c},\;\mathrm{in}\;0<\zeta <1, & (14b) \end{array}$$

with the following initial and boundary conditions:

$$\begin{array}{c} c\left(\zeta ,0\right)=c_{0},0<\zeta <1,\\ c\left(\zeta ,\tau_{c}^{-}\right)=c\left(\zeta ,\tau_{c}^{+}\right),0<\zeta <1,\\ \frac{\partial c}{\partial \zeta }\left(0,\tau \right)=0,\tau >0,\\ c(1,\tau )=0,\tau >0. \end{array}$$

This model is subject to the initial and boundary conditions, as expressed in Equation (5), with the addition of a continuity condition, $c\left(\zeta ,\tau_{c}^{-}\right)=c\left(\zeta ,\tau_{c}^{+}\right)$. The continuity condition is introduced to ensure a continuous drug release from both phases at $t_{c}$. We assumed that the solution for the first phase of the model is given by the solution of the one-phase model, Equation (13) with $D=D_{0}$ at $0<t<t_{c}$. Thus, we proceed to solve the second phase model, Equation (14b).

Again, the separation of variables method is used to solve Equation (14b). Two sets of solution were obtained for the ODE by applying the fundamental steps for the separation of variables method, which are

$$A\left(\zeta \right)=a_{2}\cos\frac{\left(2n+1\right)}{2}\pi \zeta$$

and

$$B\left(\tau \right)=\frac{CX(\tau_{c})}{X(\tau )}e^{-D_{1}{\left(\frac{2n+1}{2}\pi \right)}^{2}\int_{\tau_{c}}^{\tau }X{\left(t\right)}^{-2}dt}.$$

The solution for the space-dependent function, A(ζ), is the same as that discussed in Section 2.1 because the conditions are the same throughout the device, 0<ζ<1. However, the solution for the time-dependent function, B(τ), differs because we consider drug diffusion at the second phase, which begins at $\tau =\tau_{c}$ instead of at τ=0. Then, by using superposition, we obtain the following solution:

(15) $$c\left(\zeta ,\tau \right)=\sum_{n=0}^{\infty }h_{n}\frac{X(\tau_{c})}{X(\tau )}\cos\left(\frac{\left(2n+1\right)}{2}\pi \zeta \right)e^{-D_{1}{\left(\frac{2n+1}{2}\pi \right)}^{2}\int_{\tau_{c}}^{\tau }X{\left(t\right)}^{-2}dt},\tau \geq \tau_{c}.$$

To determine $h_{n}$, we apply the continuity condition at $\tau =\tau_{c}$, and the coefficients obtained from both equations are matched. From the continuity condition, $c(\zeta ,\tau_{c}^{-})$ and $c(\zeta ,\tau_{c}^{+})$ represent the drugs’ concentration in the hydrogel at $\tau_{c}$ in the first and second phases, respectively. Function $c(\zeta ,\tau_{c}^{-})$ is taken from Equation (9) with $\tau =\tau_{c}$ and $D=D_{0}$ while function $c(\zeta ,\tau_{c}^{+})$ is taken from Equation (15) with $\tau =\tau_{c}$.

$$c\left(\zeta ,\tau_{c}^{-}\right)=c\left(\zeta ,\tau_{c}^{+}\right)$$

$$\begin{array}{c} \frac{4}{\pi }c_{0}\sum_{n=0}^{\infty }\frac{{\left(-1\right)}^{n}}{\left(2n+1\right)}\frac{L}{X(\tau_{c})}\cos\left(\frac{2n+1}{2}\pi \zeta \right)e^{-D_{0}{\left(\frac{2n+1}{2}\pi \right)}^{2}\int_{0}^{\tau_{c}}X{\left(t\right)}^{-2}dt}=\\ \sum_{n=0}^{\infty }h_{n}\frac{X(\tau_{c})}{X(\tau_{c})}\cos\left(\frac{2n+1}{2}\pi \zeta \right)e^{-D_{1}{\left(\frac{2n+1}{2}\pi \right)}^{2}\int_{\tau_{c}}^{\tau }X{\left(t\right)}^{-2}dt}. \end{array}$$

The coefficient $h_{n}$ obtained is

$$h_{n}=\frac{4}{\pi }c_{0}\frac{{\left(-1\right)}^{n}}{\left(2n+1\right)}\frac{L}{X(\tau_{c})}e^{-D_{0}{\left(\frac{2n+1}{2}\pi \right)}^{2}\int_{0}^{\tau_{c}}X{\left(t\right)}^{-2}dt}.$$

The coefficient $h_{n}$ is then substituted into Equation (15), giving

(16) $$\begin{array}{c} c\left(\zeta ,\tau \right)=\frac{4}{\pi }c_{0}\sum_{n=0}^{\infty }\frac{{\left(-1\right)}^{n}}{\left(2n+1\right)}\frac{L}{X(\tau )}\cos\left(\frac{2n+1}{2}\pi \zeta \right)e^{-{\left(\frac{2n+1}{2}\pi \right)}^{2}\left[D_{0}\left(\int_{0}^{\tau_{c}}X^{-2}dt\right)+D_{1}\left(\int_{\tau_{c}}^{\tau }X{\left(t\right)}^{-2}dt\right)\right]}. \end{array}$$

This solution gives an equation of concentration of drugs in the hydrogel in the second phase. Further, we substitute Equation (16) into Equation (10) to solve for the fractional drug release in a swelling device after the critical time.

$$\begin{array}{cc} R\left(\tau \right)=\frac{M(\tau )}{M(0)} & =1-\frac{X(\tau )}{Lc_{0}}\int_{0}^{1}c(\zeta ,\tau )d\zeta \\ \; & =1-\sum_{n=0}^{\infty }\frac{8}{{\left(2n+1\right)}^{2}\pi^{2}}e^{-{\left(\frac{2n+1}{2}\pi \right)}^{2}\left[D_{0}\left(\int_{0}^{\tau_{c}}X{\left(t\right)}^{-2}dt\right)+D_{1}\left(\int_{\tau_{c}}^{\tau }X{\left(t\right)}^{-2}dt\right)\right]}. \end{array}$$

In the original variable, the function for fractional drug release on the second phase is

(17) $$R\left(t\right)=1-\sum_{n=0}^{\infty }\frac{8}{{\left(2n+1\right)}^{2}\pi^{2}}e^{-{\left(\frac{2n+1}{2}\pi \right)}^{2}\left[D_{0}\left(\int_{0}^{t_{c}}X{\left(t^{\prime }\right)}^{-2}dt^{\prime }\right)+D_{1}\left(\int_{t_{c}}^{t}X{\left(t^{\prime }\right)}^{-2}dt^{\prime }\right)\right]},\;t\geq t_{c}.$$

Similarly, for this two-phase model, we consider the logistic growth function, Equation (12), to represent the swelling function, X(t). Therefore, the integral terms in Equation (17) can be further specified as

$$\int_{0}^{t_{c}}X{\left(t^{\prime }\right)}^{-2}dt^{\prime }=\frac{1}{2L^{2}m^{2}g}\left[{\left(m-1\right)}^{2}\left(1-e^{-2gt_{c}}\right)+4\left(m-1\right)\left(1-e^{-gt_{c}}\right)+2gt_{c}\right]$$

and

$$\int_{t_{c}}^{t}X{\left(t^{\prime }\right)}^{-2}dt^{\prime }=\frac{1}{2L^{2}m^{2}g}\left[{\left(m-1\right)}^{2}\left(e^{-2gt}-e^{-2gt_{c}}\right)+4\left(m-1\right)\left(e^{-gt}-e^{-gt_{c}}\right)+2g\left(t-t_{c}\right)\right].$$

To simplify the equations, let

$$\int_{0}^{t_{c}}X{\left(t^{\prime }\right)}^{-2}dt^{\prime }=\frac{1}{2L^{2}m^{2}g}P\left(t_{c}\right),$$

and

$$\int_{t_{c}}^{t}X{\left(t^{\prime }\right)}^{-2}dt^{\prime }=\frac{1}{2L^{2}m^{2}g}Q\left(t\right),$$

where

$$P\left(t_{c}\right)={\left(m-1\right)}^{2}\left(1-e^{-2gt_{c}}\right)+4\left(m-1\right)\left(1-e^{-gt_{c}}\right)+2gt_{c},$$

and

$$Q\left(t\right)={\left(m-1\right)}^{2}\left(e^{-2gt_{c}}-e^{-2gt}\right)+4\left(m-1\right)\left(e^{-gt_{c}}-e^{-gt}\right)+2g\left(t-t_{c}\right).$$

Thus, Equation (17) can then be expressed as

(18) $$R\left(t\right)=1-\sum_{n=0}^{\infty }\frac{8}{{\left(2n+1\right)}^{2}\pi^{2}}e^{-\frac{1}{2g}{\left(\frac{2n+1}{2Lm}\pi \right)}^{2}\left[D_{0}\left(P\left(t_{c}\right)\right)+D_{1}\left(Q\left(t\right)\right)\right]},\;t\geq t_{c}.$$

Finally, placing the solutions of fractional drug release from both phases together gives

(19) $$R\left(t\right)=\left\{\begin{array}{c} 1-\sum_{n=0}^{\infty }\frac{8}{{\left(2n+1\right)}^{2}\pi^{2}}e^{-\frac{D_{0}}{2g}{\left(\frac{2n+1}{2Lm}\pi \right)}^{2}P\left(t\right)},0<t\leq t_{c},\\ 1-\sum_{n=0}^{\infty }\frac{8}{{\left(2n+1\right)}^{2}\pi^{2}}e^{-\frac{1}{2g}{\left(\frac{2n+1}{2Lm}\pi \right)}^{2}\left[D_{0}\left(P\left(t_{c}\right)\right)+D_{1}\left(Q\left(t\right)\right)\right]},t\geq t_{c}. \end{array}\right.$$

Therefore, the newly established two-phase model, which considers the dynamic diffusion coefficient is complete.

### 2.3. Algorithm for the Two-Phase Model

To determine the growth parameter, *g*, diffusion coefficients, $D_{0}$ and $D_{1}$, and critical time, $t_{c}$, we implement the least-squares method in the two-phase model. The numerical algorithm for the method is as follows:

Step 1 Input the experimental data with discrete value i=1,2,3,…..,K.
  Time, $t_{i}$: ${\bar{t}}_{1},{\bar{t}}_{2},{\bar{t}}_{3},\ldots ..,{\bar{t}}_{K}$.
  Devices’ radius, $\bar{X}\left(t_{i}\right)$: $\bar{X}\left(t_{1}\right),\bar{X}\left(t_{2}\right),\bar{X}\left(t_{3}\right),\ldots ..,\bar{X}\left(t_{K}\right)$.
  Fractional drug release, $\bar{R}\left(t_{i}\right)$: ${\bar{R}}_{1},{\bar{R}}_{2},{\bar{R}}_{3},\ldots ..,{\bar{R}}_{K}$.
Step 2 Find the determined growth parameter, $g^{*}$, such that it minimises
  $$\varepsilon \left(g\right)=\sum_{j=1}^{K}{\left(\bar{X}\left(t_{j}\right)-X\left(t_{j},g\right)\right)}^{2},$$
  where function $\bar{X}\left(t_{j}\right)$ represents the devices’ radius at time $t_{j}$ and $X(t_{j},g)$ is the logistic function defined in Equation (12) using growth parameter *g* at time $t_{j}$.
Step 3 Output the determined growth parameter, $g^{*}$. Use $g=g^{*}$ for the next step.
Step 4 Find $\left(D_{0}^{*},D_{1}^{*}\right)$ such that it minimises
  $$\varepsilon \left(t_{k},D_{0},D_{1}\right)=\sum_{k=1}^{K}{\left(\bar{R}\left(t_{k}\right)-R_{N}\left(t_{k},t_{c},D_{0},D_{1}\right)\right)}^{2},$$
  where $\bar{R}\left(t_{k}\right)$ represents the fractional drug release data and $R_{N}$ is
  $$R_{N}\left(t,t_{c},D_{0},D_{1}\right)=\left\{\begin{array}{c} 1-\sum_{n=0}^{N}\frac{8}{{\left(2n+1\right)}^{2}\pi^{2}}e^{-\frac{D_{0}}{2g}{\left(\frac{2n+1}{2Lm}\pi \right)}^{2}P\left(t\right)},0<t\leq t_{c},\\ 1-\sum_{n=0}^{N}\frac{8}{{\left(2n+1\right)}^{2}\pi^{2}}e^{-\frac{1}{2g}{\left(\frac{2n+1}{2Lm}\pi \right)}^{2}\left[D_{0}\left(P\left(t_{c}\right)\right)+D_{1}\left(Q\left(t\right)\right)\right]},t\;\geq t_{c}, \end{array}\right.$$
  where
  $$P\left(t\right)={\left(m-1\right)}^{2}\left(1-e^{-2gt_{c}}\right)+4\left(m-1\right)\left(1-e^{-gt_{c}}\right)+2gt_{c},$$
  $$P\left(t_{c}\right)={\left(m-1\right)}^{2}\left(1-e^{-2gt_{c}}\right)+4\left(m-1\right)\left(1-e^{-gt_{c}}\right)+2gt_{c},$$
  and
  $$Q\left(t\right)={\left(m-1\right)}^{2}\left(e^{-2gt}-e^{-2gt_{c}}\right)+4\left(m-1\right)\left(e^{-gt}-e^{-gt_{c}}\right)+2g\left(t-t_{c}\right).$$
  If $\varepsilon \left(t_{k},D_{0}^{*},D_{1}^{*}\right)<\varepsilon_{opt}$, then $\varepsilon_{opt}=\varepsilon \left(t_{k},D_{0}^{*},D_{1}^{*}\right)$, $D_{opt}=\left(D_{0}^{*},D_{1}^{*}\right)$ and $t_{c}=t_{k}$.
Step 5 Output the optimal parameters, $\varepsilon_{opt}$, $D_{opt}$, and $t_{c}$, and stop.

## 3. The Experiment

Three different formulations of stimuli-responsive bacterial cellulose-*g*-poly(acrylic acid) (BC-*g*-P(AA)) hydrogels were prepared, as reported earlier [9]. Briefly, three dispersions of 1% bacterial cellulose (BC) and acrylic acid (AA) were prepared by mixing them at 20:80, 30:70, and 40:60. These dispersions were then irradiated with electron beam (EB) at 35 kGy irradiation dose and named as 208035, 307035, and 406035 hydrogels. The BC/AA ratio for 208035, 307035, and 406035 hydrogels are 20% AA and 80% of 1% BC, 30% AA and 70% of 1% BC, and 40% AA and 60% of 1% BC, respectively. Thereafter, EB synthesised hydrogels were extracted by repeated immersion in deionised water for seven days, dried, and then cut into discs of 1 cm diameter.

### 3.1. Drug Loading

Bovine serum albumin (BSA), a readily water-soluble model protein drug, was physically loaded (entrapped) into the hydrogel discs by the swelling diffusion method, as described earlier [9]. Briefly, discs were soaked into 25 mL of BSA solution for 24 h at 37 °C. The hydrogel discs were then rinsed with 0.1 M HCl and dried at 25 °C. The concentration of BSA in the loading solution before and after the experiment was determined using Bradford reagent assay. The entrapment efficiency (%) of the BSA in hydrogels was calculated to be 54.5%, 44.4%, and 39.7% for 208035, 307035, and 406035 hydrogels, respectively.

### 3.2. Drug Release

In vitro release experiments were performed by placing the hydrogels into the simulated gastric fluid (SGF) with pH 1.2 for the first 2 h, and then immediately transferred into the simulated intestinal fluid (SIF) with pH 6.8 for the remaining time until maximum drug release. This experiment was done at 37 °C with constant shaking at 50 rpm. The growth of hydrogel and the concentration of the drug in both fluids were measured on an hourly basis. After each hour, 20 mL samples were drawn from release media and Bradford assay was performed to determine the amount of BSA released.

## 4. Results and Discussion

The numerical test of the newly developed mathematical model was performed on the experimental data of the three different formulations of hydrogels. The results of our previous study [9] suggest that the drug release in the SGF was negligible because of the lower swelling of hydrogels in acidic pH.

Hence, only the experimental data in SIF were employed for numerical analysis of the developed models. The diffusion and swelling processes are assumed to be uniform and occur only in the radial direction. Thus, the 1D region that we consider is from the centre of the disc (x=0) to the tip of the device’s radius (x=X(t)), as shown in Figure 2.

Both of the newly developed fractional drug release models, that is the one-phase model, Equation (13), and the two-phase model, Equation (19), were tested with the experimental data of the three different formulations of swelling hydrogel-based delivery devices. Parameters *g*, $D_{0}$, $D_{1}$, and $t_{c}$ were estimated for each hydrogel using the LSQCURVEFIT function in MATLAB software.

### 4.1. Model Test for Device Swelling

First, the growth parameter, *g*, for all hydrogels was determined using the logistic growth function. The logistic growth function, X(t), represents the radius of the drug device at time *t*. The logistic function shows an increase in the radius of the drug devices from the original length until it reaches the maximum length.

**Figure 3:**
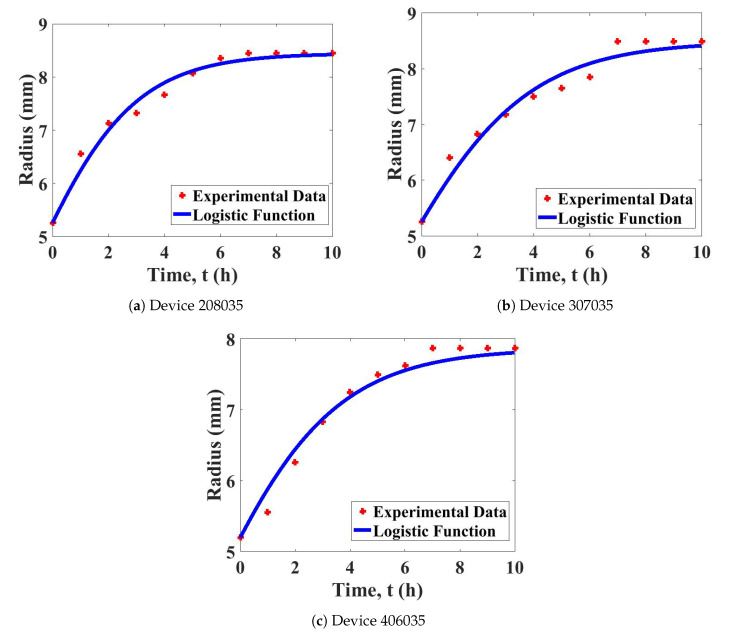
Fitted curve of the logistic growth model with the experimental data for the hydrogels: (**a**) 208035; (**b**) 307035; and (**c**) 406035.

Figure 3 shows that the swelling behaviour of all devices is well-fitted with the logistic function. As presented in Table 1, the determined growth parameter value of the 208035 hydrogel is the highest of all tested devices. This result shows that the 208035 hydrogel swells faster than the other two devices. Meanwhile, the determined growth parameters of the other two hydrogels (307035 and 406035) were similar.

### 4.2. Model Test on Drug Release Profiles

In this section, the one-phase model and the two-phase models are fitted to the experimental data, and the results are compared. Parameters $D_{0}$, $D_{1}$, and $t_{c}$ were determined in this process.

**Figure 4:**
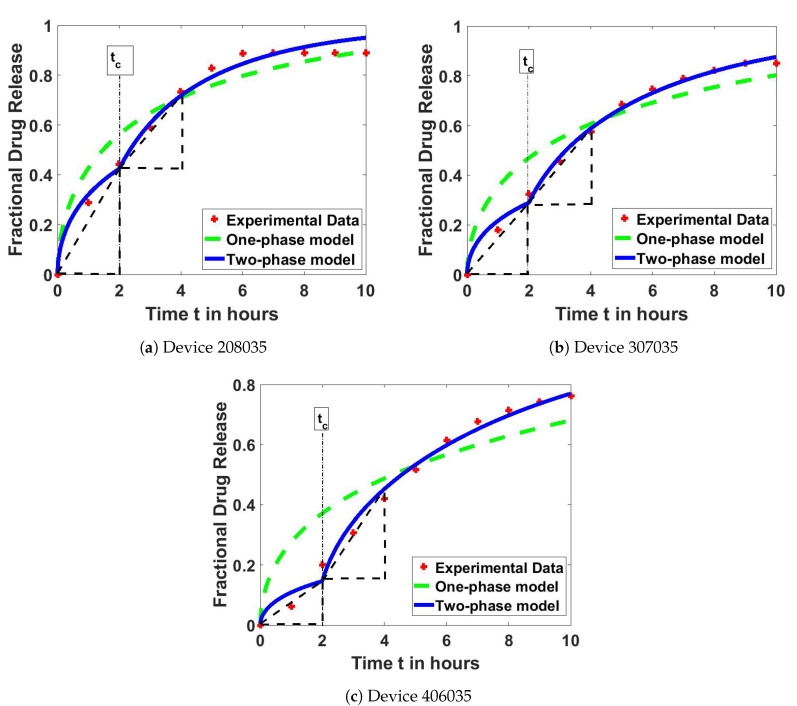
Fitted curve of the fractional drug release models, which are the one-phase and two-phase models, with the experimental data of the hydrogels: (**a**) 208035; (**b**) 307035; and (**c**) 406035.

Figure 4 shows that, for all tested hydrogels, the curve-fitting of the extended two-phase model fits much better than the basic one-phase model. The diffusion coefficients of both models are determined and presented in Table 2. These results suggest that the determined diffusion coefficient in both models (*D*, $D_{0}$, and $D_{1}$) have a descending order for the 208035, 307035, and 406035 hydrogels, thereby indicating that the drug release is the fastest in the 208035 hydrogel among the three tested hydrogels. This result is closely related to the pore size of the tested hydrogels. According to our previous findings [9], the pore size of the 208035 hydrogel is highest (i.e., hydrogels are less dense) as compared to the other two hydrogels where the pore size of 208035, 307035, and 406035 hydrogels are around 50–100, 12–27, and 4–14 μm, respectively.

Table 2 presents that the determined diffusion coefficients of all tested hydrogels are lower in the first phase than in the second phase ($D_{0}<D_{1}$), where $D_{0}$ is about 70–90% slower than $D_{1}$. This condition is because of the increase in the pore size of the devices as they swell, thereby resulting in easier and faster diffusion. Furthermore, the determined diffusion coefficient *D* of the one-phase model is in between the values of diffusion coefficients $D_{0}$ and $D_{1}$ of the two-phase model.

Although the 208035 hydrogel has lower diffusivity in the first phase ($D_{0}<D_{1}$), it appears to release drugs more quickly in the initial phase. As shown in Figure 4b, the steeper slope in the first phase of the two-phase model curve indicates a higher initial fractional drug release rate from the device. The fractional drug release rate can be calculated by

$$\frac{\Delta R}{\Delta t}=\frac{R_{i+h}-R_{i}}{t_{i+h}-t_{i}},$$

where *R* is the fractional drug release, *t* is the time, and *h* is the step size. If the fractional drug release rate in the first phase is higher than the second phase (${\frac{\Delta R}{\Delta t}|}_{t<t_{c}}>{\frac{\Delta R}{\Delta t}|}_{t\geq t_{c}}$), we can say that the drug device releases drugs more quickly initially. As presented in Table 3, the measured value of $\frac{\Delta R}{\Delta t}$ of the 208035 hydrogel is higher in the first phase than the second phase (${\frac{\Delta R}{\Delta t}|}_{t<t_{c}}>{\frac{\Delta R}{\Delta t}|}_{t\geq t_{c}}$), thereby demonstrating the fast initial drug release. Since $D_{0}<D_{1}$ for 208035 hydrogel, it shows that this condition is not caused by the diffusivity.

Based on the results in Table 1, we found that the 208035 hydrogel gives the highest value of the determined growth parameter. In our previous study [9], we observed that hydrogels with greater swelling exhibited higher entrapment efficiency, which means that hydrogel with a higher growth rate has more drugs loaded at the beginning. The high drug load might lead to a fast initial drug release rate.

Nevertheless, the resulted curve fitting of the two-phase model improved significantly as compared to the basic one-phase model. This finding is supported by the error values listed in Table 4. The 208035 hydrogel showed the least error improvement as compared to the other two devices. The least-squares error of the two-phase model for the 307035 and 406035 hydrogels were almost ten times smaller than that of the one-phase model. Nonetheless, this new two-phase model manages to reduce the least-squares error for all the tested drug devices.

## 5. Conclusions

In this study, we successfully developed a new two-phase drug release model considering device swelling. The performance of the developed two-phase model was numerically tested using the experimental data of three different BC-g-P(AA) hydrogels. It is shown that the newly developed two-phase model exhibited significant improvement to the previously known model of drug release in a swelling device. The least-squares error was reduced, and the established model proved to be capable of estimating the drug release in a swelling drug delivery device. The numerical results also detected that the initial diffusion coefficients for all three hydrogels were lower than the later diffusion coefficients. The smaller diffusion coefficient at the initial phase as compared to the later phase was mainly related to the changes in the device pore size because of swelling. A detected initial burst for device 208035 is believed to be caused by the high growth parameter of that device, which leads to a high amount of drug loaded. The parameters of this model helped in analysing and predicting the behaviour of a swelling drug delivery device. Thus, this model will facilitate the researcher in designing controlled drug delivery devices in the future. This model can be used to determine the parameters of drug release kinetics from a swellable matrix system using the in vitro results.

## Figures and Tables

**Table 1:** Determined value of the growth parameter.

Device	Growth Parameter,	Least-Squares Error
	g	
208035	1.49918 $\times \;10^{-4}$	0.27483
307035	1.17298 $\times \;10^{-4}$	0.44279
406035	1.16996 $\times \;10^{-4}$	0.25559

**Table 2:** Determined value of diffusion coefficients and critical time.

Devices	One-Phase Model	Two-Phase Model		
	D	$D_{0}$	$D_{1}$	$t_{c}$
	**(mm** ${}^{2}$ **/s)**	**(** ${\mathrm{mm}}^{2}/s$ **)**	**(** ${\mathrm{mm}}^{2}/s$ **)**	**(s)**
208035	1.30515 $\times \;10^{-3}$	7.37805 $\times \;10^{-4}$	2.22787 $\times \;10^{-3}$	7200
307035	8.58865 $\times \;10^{-4}$	3.25908 $\times \;10^{-4}$	1.49489 $\times \;10^{-3}$	7200
406035	5.10114 $\times \;10^{-4}$	7.90305 $\times \;10^{-5}$	9.35150 $\times \;10^{-4}$	7200

**Table 3:** Measured values of the fractional drug release rate.

Device	$\frac{\Delta R}{\Delta t}$	$\frac{\Delta R}{\Delta t}$
	($0\leq t\leq t_{c}$)	($t\geq t_{c}$)
208035	0.20984	0.14669
307035	0.14255	0.14895
406035	0.07102	0.15310

**Table 4:** Least-squares error.

Device	One-Phase Model	Two-Phase Model
208035	0.06429	0.01237
307035	0.08800	0.00477
406035	0.13910	0.01015
